# Brain Circuits Mediating the Orexigenic Action of Peripheral Ghrelin: Narrow Gates for a Vast Kingdom

**DOI:** 10.3389/fendo.2015.00044

**Published:** 2015-03-30

**Authors:** Agustina Cabral, Pablo N. De Francesco, Mario Perello

**Affiliations:** ^1^Laboratory of Neurophysiology, Multidisciplinary Institute of Cell Biology [Argentine Research Council (CONICET) and Scientific Research Commission, Province of Buenos Aires (CIC-PBA)], Buenos Aires, Argentina

**Keywords:** food intake, appetite, brain accessibility

The nervous and endocrine systems act together to regulate all physiological processes essential for the body homeostasis control. Given the strict communication restrictions that the brain–blood barrier (BBB) imposes, the interplay between these two systems requires a variety of delicate anatomical interfaces and physiological mechanisms that guarantee the precise function of the neuroendocrine system as a whole. The study of the mechanisms by which hormones act in the brain in order to regulate specific neuronal populations is a research topic rather neglected. Our group studies the neuronal circuitries and molecular mechanisms by which the stomach-produced hormone ghrelin regulates appetite and other physiological functions. A clear notion of the brain targets of peripheral ghrelin is essential for the comprehensive understanding of the physiological role of this hormone. Ghrelin is called “the hunger hormone” since it is the only known orexigenic peptide hormone. The target for ghrelin orexigenic actions is the brain, which contains a variety of ghrelin-responsive nuclei; however, several evidences suggest that the accessibility of peripheral ghrelin to the brain is strikingly low. Here, we briefly summarize the current knowledge in this topic and discuss this intriguing neuroendocrinological issue.

Ghrelin is a 28-amino acid octanoylated peptide predominantly secreted from endocrine cells located within the gastric mucosa (1). Ghrelin acts via the growth hormone secretagogue receptor 1A (GHSR-1A), a G-protein-coupled receptor highly expressed in the brain (2, 3). Ghrelin’s central actions include modulation of the growth hormone secretion, blood glucose homeostasis, stress responses, and gastrointestinal tract motility, among others (4). Notably, ghrelin is recognized as the only mammalian peptide hormone able to increase appetite (5, 6). Ghrelin orexigenic effects are rapid, since food intake increases within 5–10 min after its systemic administration (7). Thus, the ghrelin brain’s accessibility must be relevant for this unique role of the hormone. Direct ghrelin micro-injections, ranging from 10 to 800 pmol, in several brain areas potently increase food intake (6). Ghrelin-induced food intake recruits neuronal circuits, located in the hypothalamus and the brainstem, which regulate appetite depending on the energy store levels (6). In particular, ghrelin orexigenic actions depends on the hypothalamic arcuate nucleus (ARC), which highly expresses GHSR-1A and is located in close apposition to the median eminence (ME), an important circumventricular organ (3, 8). Ghrelin-induced food intake also occurs at other GHSR-1A-expressing hypothalamic areas that lack obvious access to circulating ghrelin, such as the paraventricular nucleus, the lateral hypothalamus, and the ventromedial nucleus (9–11). Ghrelin orexigenic actions also take place at the dorsal–vagal complex, which expresses GHSR-1A and includes the nucleus of the solitary tract, the dorsal motor nucleus, and the area postrema (AP), another important circumventricular organ (3, 12). In addition, ghrelin regulates appetite and some rewarding aspects of eating by directly acting on the ventral tegmental area and other centers of the mesolimbic pathway, which also express GHSR-1A (13, 14). Thus, the ghrelin-induced food intake depends on the ability of peripheral ghrelin to impact on these distributed brain targets.

Peptide hormones cannot freely enter the brain. The BBB displays specific transport mechanisms that can serve for particular peptides to gain access into the brain through an otherwise impermeable boundary. This transport mechanism can include (a) receptor-mediated transcytosis, (b) non-specific binding of positively charged peptides to the cell membrane and further transcytosis, (c) diffusion of molecules through the so-called extracellular pathway, or (d) free transmembrane diffusion, as seen for some small peptides (15). The fenestrations of the specialized capillaries of circumventricular organs allow for small peptides to penetrate the intercellular space and eventually diffuse toward neighboring brain areas (16). Notably, the ventromedial ARC represents an exceptional case of privileged permeability to blood-borne factors due to presence of a fenestrated vasculature branching from the ME (17). Peptide hormones can also reach the brain via the cerebrospinal fluid (CSF) after crossing the blood–CSF barrier at either the choroids plexus, a specialized layer of cuboidal ependymal cells that surround a core of capillaries in some brain ventricles and produce the CSF, and/or the hypothalamic tanycytes, a specialized layer of bipolar ependymal cells that line the floor of the third ventricle and bridge the CSF and the capillaries of the ME (18, 19). Additionally, some hormones signal to the brain by acting on sensory circumventricular organs, such as the subfornical organ or the AP, which detect plasma hormone levels and transmit such information into specific brain regions (20). Currently, little is known in terms of which of these mechanisms underlie central orexigenic ghrelin actions.

In order to clarify the brain areas mediating orexigenic effects of peripheral ghrelin, we have recently performed a detailed neuroanatomical analysis in mice of both the distribution of ghrelin-induced increase of the marker of cellular activation c-Fos and the brain areas accessible to fluorescent ghrelin (8). We used a high (0.6 nmol/g BW) and a low (0.06 nmol/g BW) doses of ghrelin that induce a ~17- and 2-fold increase of plasma ghrelin concentrations 30 min after treatment, respectively. Interestingly, we found that the smaller increment of circulating ghrelin, which is sufficient to increase food intake, exclusively impacts the ARC/ME while the higher increment of circulating ghrelin increases c-Fos expression in and access not only the ARC but also the AP. In addition, the higher increment of circulating ghrelin accesses the periventricular hypothalamic regions and induces c-Fos expression in a few extra-brain areas such as the paraventricular nucleus and the nucleus of the solitary tract. Notably, fluorescent ghrelin was detected in tanycyte-like cells of the ME in all mice peripherally treated with the tracer. Ghrelin internalization in tanycytes has been recently confirmed *in vivo* and shown to also occur *in vitro* (21). Importantly, we showed that centrally administered ghrelin reaches and increases c-Fos levels in most of the GHSR-1A-expressing brain areas (8, 22). In addition, we found that ARC-ablated mice fail to eat in response to peripherally administered ghrelin but fully respond to the orexigenic effects of the centrally administered hormone. Thus, our data support the notion that the ARC is the main target of the orexigenic effects of peripheral ghrelin in mice.

Our study stresses the fact that the accessibility of peripheral ghrelin to the brain is surprisingly limited and restricted to specific brain areas. Figure 1 summarizes our observations in terms of the brain accessibility of peripherally administered fluorescent ghrelin, and describes the potential mechanisms mediating this phenomenon. A limited ghrelin brain accessibility is in line with a seminal study showing that radioactive ghrelin is transported across the BBB in the brain-to-blood direction by a saturable system while blood-to-brain influx is extremely low (23). In addition, very limited amounts of peripherally injected ghrelin can be detected in the CSF of ewes (24). Our study adds neuroanatomical insights to these reports by showing that the ghrelin accessibility mainly occurs at the ARC/ME and, to a lesser extent, the AP. Thus, peripheral ghrelin seems to mainly reach and activate brain areas located close to some circumventricular organs, which would allow the access of the circulating hormone to the brain. In this regard, a recent study has shown that ghrelin passively and rapidly extravasates through fenestrated capillaries of the ME and reaches nearby brain regions (16). A higher accessibility at the ARC/ME has been also shown for other peptide hormones, including insulin and leptin (25, 26). Peripheral ghrelin could impact on specific neuronal circuits by acting at the subfornical organ, which expresses GHSR-1A; however, this pathway is not related to food intake regulation (20). The presence of fluorescent ghrelin in both the tanycytes and the choroids plexus (unpublished observation) and the hypothalamic periventricular regions of peripherally injected mice suggest a potential ghrelin transport from the periphery to the CSF that could impact on food intake regulation. Interestingly, it has been proposed that tanycytic could take up circulating leptin from the ME and transport it toward the apical cell pole in contact with the CSF (27). Further studies are required in order to test if ghrelin can access the brain via this mechanism. Notably, the different profile of c-Fos induction and fluorescent ghrelin distribution found for the lower and higher doses of ghrelin may indicate that different mechanisms of entry to the brain can take place depending on plasma hormone levels. The observation that very high increases in plasma ghrelin level are not sufficient to access and/or activate deeper brain areas known to express GHSR-1A support the notion that the transport of ghrelin through the BBB in a blood-to-brain direction is extremely limited in mice. Importantly, our study was performed using a single bolus of fluorescent ghrelin and 15 min after injection. Thus, our data do not invalidate that ghrelin may act on other brain areas through slower mechanisms either after a sustained increase of the hormone concentration or over prolonged time periods. However, these potential slow ghrelin-responsive pathways unlikely mediate the rapid orexigenic effects of the hormone. Interestingly, a recent article reported that mice fail to increase food intake in response to 0.0075 nmol/g BW of ghrelin, which increases ~14-fold plasma ghrelin concentrations 10 min after treatment, and concluded that supraphysiologic plasma ghrelin levels are required in order to stimulate appetite (28). The lack of an orexigenic response may be related to the transient nature of the ghrelin peak induced in these conditions, given the short half-life of the hormone (29). Still, these data also stress the limited brain response, in terms of food intake, to huge increases in plasma ghrelin levels.

**Figure 1:**
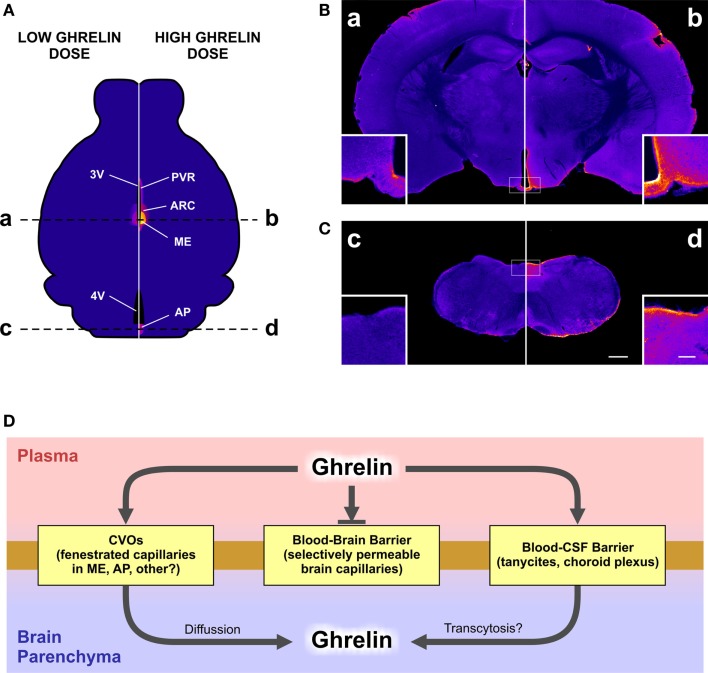
**The figure depicts the differential brain accessibility of a high or a low dose of peripherally administered ghrelin and the potential mechanisms mediating ghrelin brain accessibility**. In our study, a set of mice were subcutaneously injected with a high (0.6 nmol/g BW) or a low (0.06 nmol/g BW) dose of fluorescent ghrelin and perfused 15 min later. Brains were removed, post-fixed, cryoprotected, and coronally cut. The neuroanatomical mapping of the presence of the ghrelin tracer was performed by the analysis of the amplified fluorescein-immunoreactive signal achieved by an anti-fluorescein antibody followed by biotinylated secondary antibody, streptavidin–peroxidase, and a chromogenic reaction. Stained brain sections were mounted and bright-field images were acquired with a digital camera at 16 bit/pixel. Finally, pixel intensity values were converted into optical density data by taking the negative decimal logarithm of the original value divided by the white flat field value and pseudocolored. Yellow to blue coloring represents higher to lower levels of fluorescein-immunoreactive signal. **(A)** displays a schematic illustration representing the distribution of fluorescein signal in a mouse brain after the peripheral administration of the low (left) or the high (right) dose of the ghrelin tracer. AP, area postrema; ARC, arcuate nucleus; ME, median eminence; PVR, periventricular regions; 3V, third ventricle; 4V, fourth ventricle. **(B,C)** show the actual images of mouse coronal brain sections, at the rostro-caudal levels of the brain labeled with dashed lines in the **(A)**. Letters a, b, c, and d label corresponding brain levels and ghrelin doses between **(A)** and **(B,C)**. Insets in each microphotography show higher magnification images. Scale bars: 500 μm in low magnification, 100 μm in high magnification. **(D)** displays a schematic diagram of the potential mechanisms mediating the ghrelin brain accessibility. CVOs, circumventricular organs; CSF, cerebrospinal fluid.

Overall, it seems clear that most of the neuronal circuits known to regulate food intake are sensitive to ghrelin. However, the physiological relevance of the peripheral ghrelin signaling on these targets is unclear given the limited brain accessibility of hormone. The possibility that some of these neuronal circuits are engaged by centrally produced ghrelin has been disregarded since it seems now clear that ghrelin is not synthesized in the brain (30, 31). One possibility is that the ghrelin brain accessibility could be regulated under particular physiological states. Indeed, the extent to which the diffusion of molecules in the ARC/ME occurs can be regulated in circumstances, such as fasting, by modulating the amount of vascular fenestrations as well as by reorganizing the structure of the tight-junctions between tanycytes and limiting the diffusion of molecules to the CSF (32). In terms of ghrelin, it has been shown that the rate at which this hormone is transported into the brain is reduced in physiological states, such as obesity or neonatal overnutrition (21, 33). It has been also proposed that GHSR-1A can act in a ghrelin-independent manner since this receptor is able to both signal in the absence of its ligand (34) and heterodimerize with other G-protein-coupled receptors in order to allosterically modulate their activity (35). Still, the impact of the constitutive GHSR-1A signaling on the orexigenic actions of the peripheral ghrelin is unclear. As usual, the more we know, the more we realize how much we do not know. Our knowledge about the ghrelin system has notably increased lately; however, many questions remain open: which are the mechanisms governing the ghrelin brain accessibility? Can the ghrelin brain accessibility be regulated? What is the physiological role of the ghrelin-responsive neuronal circuits without obvious access to peripheral ghrelin? Given its potent orexigenic effect, the ghrelin system has been perceived as a potential pharmacological target for compounds aimed to regulate appetite. Thus, we think that research efforts should be intensified in order to solve these particular issues about the ghrelin physiology.

## Conflict of Interest Statement

The authors declare that the research was conducted in the absence of any commercial or financial relationships that could be construed as a potential conflict of interest. The Associate Editor Carol F. Elias declares that, despite having collaborated with author Mario Perello, the review process was handled objectively and no conflict of interest exists.
